# A faunistic study on the leafhoppers of northwestern Iran (Hemiptera, Cicadellidae)

**DOI:** 10.3897/zookeys.496.9059

**Published:** 2015-04-16

**Authors:** Tandis Abdollahi, Ali Reza Jalalizand, Fariba Mozaffarian, Michael Wilson

**Affiliations:** 1 Department of Plant protection, Faculty of Agriculture, Islamic Azad University, Isfahan (Khorasgan) Branch, Isfahan, Iran; 2 Insect Taxonomy Research Department, Iranian Research Institute of Plant Protection, Tehran, 19395, P.O. Box 1454, Iran; 3 Department of Natural Sciences, National Museum of Wales, Cardiff, U.K.

**Keywords:** Cicadellidae, leafhoppers, fauna, Azarbaijan-e-Sharghi, Azarbaijan-e-Gharbi, Ardabil

## Abstract

The leafhopper fauna of northwestern Iran: Azarbaijan-e-Sharghi, Azarbaijan-e-Gharbi and Ardabil provinces is listed from previously published records and from our current work. Sixty-nine species are included with four species (*Mogangella straminea* Dlabola, 1957, *Doratura stylata* (Boheman, 1847), *Macrosteles sordidipennis* (Stål, 1858) and *Psammotettix seriphidii* Emeljanov, 1962) listed as new for Iran and *Balclutha punctata* (Fabricius, 1775), as a new record for the region. A distribution map of the species in northwestern Iran is given.

## Introduction

The Auchenorrhyncha consists of approximately 42000 described worldwide species which have adopted varied life habits (Larivière et al. 2010). Moreover, they play an important role in the food chains due to their high biomass in the herb layer and provide a food source for other insects and animals (Nickel 2003).

The family Cicadellidae (leafhoppers), is the largest family within the Hemiptera, with approximately 19,500 described species in more than 40 subfamilies (Oman et al. 1990). Metcalf (1962–1968) considered the leafhoppers to represent a superfamily (the Cicadelloidea) and divided them into a number of families, currently subfamilies or tribes. Following Dietrich (2005), Cicadellidae are included in the superfamily Membracoidea with the Membracidae (treehoppers). Most Cicadellidae species tend to feed from phloem fluid (except some Cicadellinae and most Typhlocybinae) (Biedermann and Niedringhaus 2009). Moreover, some species may cause both direct and indirect damage during their feeding activity, which is sometimes economically important. The most important form of indirect damage is caused by phytoplasmas and viruses, vectored mostly by Cicadellidae (Weintraub and Beanland 2006).

The earliest available record of Auchenorrhyncha from Iran is Gardenhire (1959) who recorded some species as agricultural pests. Jiri Dlabola, from the Czech Republic, studied considerable numbers of Auchenorrhyncha species from Iran in the 1970s, which led to the discovery of more than 100 new Cicadellidae species in a long series of papers (Dlabola 1974, 1977, 1980, 1981, 1982, 1983, 1985). More recently other authors have published on the fauna: Karimzadeh et al. (1998); Haghshenas and Khajeali (2000); Lashkari et al. (2009); Taghizadeh et al. (2010); Mozaffarian and Taghizadeh (2010); Mozaffarian and Emeljanov (2010); Mozaffarian et al. (2010); Mozaffarian and Sanborn (2010, 2012, 2013); Mozaffarian and Gnezdilov (2011); Gnezdilov and Mozaffarian (2011); Moazaffarian and Wilson (2011); Moosavi and Sadeghi Namaghi (2012); Mozaffarian (2012a, b); Taghizadeh (2012); Zohdi et al. (2012); Aghagoli-Marzijarani et al. (2013), Mozaffarian (2013) and Abdollahi et al. (2013, 2014). There have been a wide range of researchers who mainly focused on the Auchenorrhyncha as pests in both agricultural and forest ecosystems in Iran such as: Gharib (1966); Kheyri and Alimoradi (1969); Kheyri (1989, 1992); Rajabi and Mirzayans (1989); Behdad (1992, 1993); Khajehali et al. (2001); Nematollahi and Khajehali (2000); Yarmand et al. (2006); Aghagoli-Marzjarani et al. (2010); Taghizadeh et al. (2010) amongst others.

Northwestern Iran (the study area) covers nearly 100,503 square kilometers and consists of three provinces: Azarbaijan-e-Sharghi, Azarbaijan-e-Gharbi and Ardabil. It is located in Irano-Turanian zoogeographical region (Firouz 2005) and in the northwest plateau of Iran. It is limited between the Caspian Sea and Caspian district in the east, Caucasus mountains in the north, Anatolian Plateau and Mesopotamian region in the west and a part of Zagros, called Humid Zagros, in the south. Hence, it is expected that the fauna of this area will be influenced by the faunal elements of all mentioned regions rather than just the Iranian Plateau. The area is considered to be the crossroads of the two main mountains of Iran (Alborz and Zagros), a part of Alpine Himalayan orogenic belt (Dewey et al. 1986) with deep valleys and has a variety of altitudes from 256 m to 2896 m. It is differentiated from other parts of Iran by the highest latitude (39°40'N) and the coldest recorded temperature (-35 °C) (Hedge and Wendelbo 1978). Zarudny (1911) considered this part of Iran as a zoogeographic zone, with a fauna similar to the Caucasus. This area was also considered as a different area from other parts of Zagros by Emeljanov (1974). Hedge and Wendelbo (1978) recognized part of Iran as an endemic zone named Armeno-Kurdic due to the distribution patterns of endemic phanerogamic plants.

The aim of this research was to collect and identify the leafhoppers in northwestern Iran and to prepare a checklist as a starting point for gathering the sporadic publications and studying the fauna for the whole of Iran. A total number of 69 species belonging to 11 subfamilies are recorded.

## Material and methods

**Figure 1.**
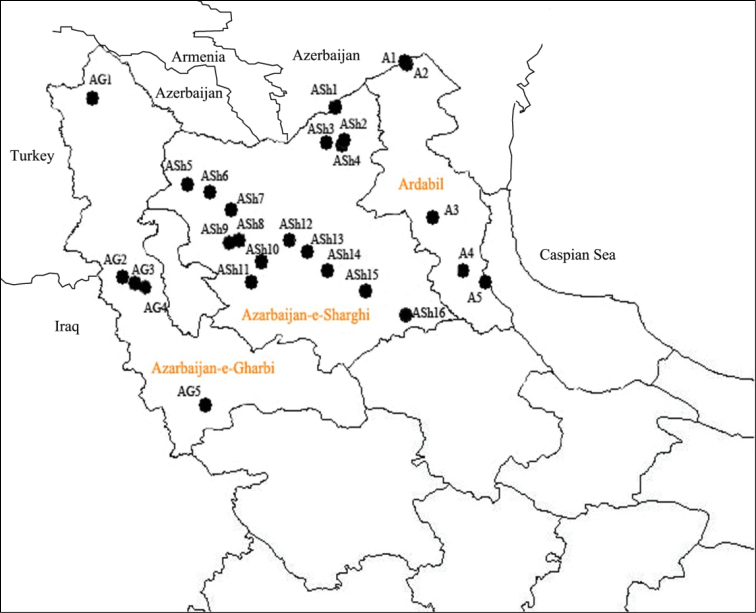
Distribution map of leafhoppers in northwestern Iran (For codes see Table 1).

The present study was carried out in three northwestern provinces including, Azarbaijan-e-Sharghi, Azarbaijan-e-Gharbi and Ardabil provinces (Figure 1). During 2007 (August, September and January) and 2008 (January) field trips were made and leafhopper specimens were collected using a sweep net. A total of 2340 specimens consists of newly collected specimens along with other specimens located in the Hayk Mirzayans Insect Museum (Tehran, Iran, which had been collected since 1968) were studied and identified. The identifications were made using the works of Le Quesne (1965, 1969), Ribaut (1952), Biedermann and Niedringhaus (2009) and Emeljanov (1997). Vouchers of all species are deposited in the Hayk Mirzayans Insect Museum. In addition to the identification, literature records were also taken into consideration and a distribution map for the leafhoppers of northwestern Iran was prepared by ARCMAP version 9.3.0.1770.

**Table 1.** List of the localities, their coordinates and the codes

Locality names	Coordinates	Locality names	Coordinates
Ajabshir	37° 35'N, 46°11'E (ASh11)	Moghan	39°37'N, 47°47'E (A1)
Bonab	37°26'N, 45°57'E (ASh9)	Eskanlu	39°12'N, 47°04'E (ASh1)
Bostanabad-Siah chaman	37°41'N, 46°59'E (ASh14)	Parsabad	39°36'N, 47°49'E (A2)
Heiran	37°41'N, 48°24'E (A4)	Sarein	38°11'N, 48°05'E (A3)
Kaleibar, Arabshahi	38°51'N, 47°08'E (ASh4)	Siah chaman-Basmenj	37°52'N, 46°46'E (ASh13)
Kaleibar, 1863m	38°52'N, 46°58'E (ASh3)	Sufian	38°16'N, 45°58'E (ASh7)
Kaleibar, 1732m	38°54'N, 47°09'E (ASh2)	Tabriz	37°58'N, 46°03'E (ASh8)
Kandovan	37°46'N, 46°17'E (ASh10)	Tabriz-Shabestar	38°15'N, 45°58'E (ASh7)
Khalkhal	37°35'N, 48°38'E (A5)	Tabriz-Bostanabad	37°58'N, 46°35'E (ASh12)
Mahabad	36°27'N, 45°42'E (AG5)	Uromieh	37°25'N, 47°42'E (AG3)
Maku	39°17'N, 44°31'E (AG1)	Uromieh, Mirzabad	37°32'N, 45°04'E (AG4)
Marand	38°25'N, 45°45'E (ASh6)	Uromieh-Sarv	37°38'N, 44°50'E (AG2)
Miyaneh-Siah chaman	37°30'N, 47°23'E (ASh15)	Zonuschay	38°29'N, 45°31'E (ASh5)
Miyaneh-Zanjan	37°17'N, 47°48'E (ASh16)		

## List of taxa

The genera and species from northwestern Iran recorded through the present study and other publications are as follows (* indicates species not found in the present study). For those species with specimens examined, a reference to the authority used for the identification is included in parenthesis following the taxon heading. The classification used follows mainly Oman et al. (1990) with changes based on more recent literature e.g., Zahniser and Dietrich (2013) for Deltocephalinae.

### Subfamily: Agalliinae

#### Tribe: Agalliini

##### Agallia firdausica

Taxon classificationAnimaliaHemipteraCicadellidae

Dlabola, 1981*

###### Localities.

Sufian (Dlabola 1981) (Fig. 1, ASh7).

###### Worldwide distribution.

Iran; Saudi Arabia (Dlabola 1980).

##### Anaceratagallia laevis

Taxon classificationAnimaliaHemipteraCicadellidae

(Ribaut, 1935)

Anaceratagallia laevis : Le Quesne 1965: 52, figs 267–268.

###### Material examined.

Azarbaijan-e-Sharghi: 1♂, 2♀, Ajabshir, Yaichi village, 1922 m, 37°35'27.2"N, 46°11'03.7"E, 15.January.2008, leg. Mozaffarian (Fig. 1, ASh11).

Dlabola (1981) reported this species from Sufian (Fig. 1, ASh7).

###### Worldwide distribution.

East Palaearctic, Europe (Albania, Britain I., Bulgaria, Canary Is., Channel Is., Cyprus, French mainland, Greek mainland, Hungary, Italian mainland, Moldova, Portuguese mainland, Romania, South Russia, Sardinia, Sicily, Spanish mainland, Ukraine, Yugoslavia), Near East, North Africa (De Jong 2013).

##### Austroagallia sinuata

Taxon classificationAnimaliaHemipteraCicadellidae

(Mulsant & Rey, 1835)

Austroagallia sinuata : Le Quesne 1965: 50, figs 253–255, 257.

###### Material examined.

Azarbaijan-e-Sharghi: 1♂, 1♀, Kaleibar, Arabshahi, 1391 m, 38°51'42.7"N, 47°08'01.1"E, 3.September.2007, leg. Mozaffarian (Fig. 1, ASh4).

Dlabola (1971, 1981) reported this species from Sufian, Maku and Miyaneh-Siah chaman (Fig. 1, Ash7, AG1, ASh15)

###### Worldwide distribution.

Afro-tropical region, East Palaearctic, Europe (Austria, Balearic Is., Belgium, Britain I., Bulgaria, Canary Is., Crete, Cyprus, Greek mainland, Hungary, Italian mainland, Moldova, Portuguese mainland, Romania, South Russia, Sardinia, Sicily, Slovakia, Spanish mainland, Switzerland, Yugoslavia), Near East, North Africa (De Jong 2013).

### Subfamily: Aphrodinae

#### Tribe: Aphrodini

##### Aphrodes bicinctus

Taxon classificationAnimaliaHemipteraCicadellidae

(Schrank, 1776)*

###### Localities.

Sufian, Marand (Dlabola 1981) (Fig. 1, ASh7, ASh6).

###### Worldwide distribution.

Europe (Albania, Austria, Belgium, Britain I., Bulgaria, Corsica, Crete, Croatia, Cyprus, Czech Republic, Danish mainland, Estonia, Finland, French mainland, Germany, Greek mainland, Hungary, Ireland, Italian mainland, Latvia, Lithuania, Madeira, Republic of Moldova, Norwegian mainland, Poland, Portuguese mainland, Romania, Russia Central, Russia North, South Russia, Sardinia, Sicily, Slovakia, Slovenia, Spanish mainland, Sweden, Switzerland, The Netherlands, Ukraine, Yugoslavia) (De Jong 2013).

### Subfamily: Cicadellinae

#### Tribe: Cicadellini

##### Cicadella viridis

Taxon classificationAnimaliaHemipteraCicadellidae

(Linnaeus, 1758)

Cicadella viridis : Le Quesne 1965: 24, fig. 115.

###### Material examined.

Ardabil: 8 ♂♀, Heiran, 1527 m, 37°41'07.4"N, 48°23'57.4"E, 18.January.2007, leg. Mozaffarian, Light trap (Fig. 1, A4).

Ardabil: 1♂, 7♀, 10 km to Parsabad, 39°36'8.3"N, 47°48'45.5"E, 18.January. 2007, leg. Mozaffarian (Fig. 1, A2).

Azarbaijan-e-Sharghi: 25 ♂♀, Eskanlu, Aras river, 290 m, 39° 12'13.4"N, 47° 04'23.2"E, 3.Sebtember.2007, leg. Mozaffarian & Nematian (Fig. 1, ASh1).

Azarbaijan-e-Gharbi: 1♂, Maku, Cheshme Soraya, 900 m, 22.August.1994, leg. Ebrahimi & Sarafrazi (Fig. 1, AG1).

Dlabola (1981) reported this species from Zonuschay (Fig. 1, ASh5).

###### Worldwide distribution.

East Palaearctic, Europe (Albania, Austria, Belgium, Britain I., Bulgaria, Croatia, Czech Republic, Danish mainland, Estonia, Finland, French mainland, Germany, Greek mainland, Hungary, Ireland, Italian mainland, Latvia, Lithuania, Moldova, Norwegian mainland, Poland, Romania, Russia Central, Russia North, South Russia, Sardinia, Sicily, Slovakia, Slovenia, Spanish mainland, Sweden, Switzerland, The Netherlands, Ukraine, Yugoslavia), Near East, Nearctic region, Oriental region (De Jong 2013).

###### Comment.

Behdad (1993) reported this species as a rice pest.

### Subfamily: Deltocephalinae

#### Tribe: Athysanini

##### Conosanus obsoletus

Taxon classificationAnimaliaHemipteraCicadellidae

(Kirschbaum, 1858)

Conosanus obsoletus : Ribaut 1952: 95, 99, figs 137–138; Le Quesne 1969: 109, figs 593, 596.

###### Material examined.

Azarbaijan-e-Gharbi: 15♂♀, Mahabad, KoushkDareh, 1499 m, 36°27'08.6"N, 045°42'32.9"E, 28.August.2007, leg. Mozaffarian & Nematian (Fig. 1, AG5).

Dlabola, 1981 reported this species from Sufian and Marand. (Fig. 1, ASh7, ASh6).

###### Worldwide distribution.

East Palaearctic, Europe (Albania, Austria, Azores, Belgium, Britain I., Bulgaria, Cyprus, Czech Republic, Danish mainland, Estonia, French mainland, Germany, Greek mainland, Hungary, Ireland, Italian mainland, Latvia, Lithuania, Moldova, Norwegian mainland, Poland, Portuguese mainland, Romania, Sicily, Slovakia, Slovenia, Spanish mainland, Sweden, Switzerland, The Netherlands, Ukraine, Yugoslavia), Near East, Nearctic region, North Africa (De Jong 2013).

##### Eohardya miyaneha

Taxon classificationAnimaliaHemipteraCicadellidae

Dlabola, 1971*

###### Localities.

Miyaneh- Siah chaman (Dlabola 1971) (Fig. 1, ASh15).

###### Worldwide distribution.

Iran (Dlabola 1971).

##### Euscelis alsius

Taxon classificationAnimaliaHemipteraCicadellidae

Ribaut, 1952

Euscelis alsius : Ribaut 1952: 95, fig. 130.

###### Material examined.

Ardabil: 2♂♀, Moghan, 65 m, 39°37'30.7"N, 47°46'57.5"E, 19.January.2007, leg. Mozaffarian (Fig. 1, A1).

Ardabil: 21♂♀, Parsabad, 80 m, 39°36'8.3"N, 47°48'45.5"E, 18.January.2007, leg. Mozaffarian (Fig. 1, A2).

Ardabil: 1♂, 1♀, 12 km to Khalkhal, 1998 m, 37°35'41.8"N, 48°37'54.3"E, 17.Janaury.2007, leg. Mozaffarian, Light trap (Fig. 1, A5).

Azarbaijan-e-Sharghi: 74♂♀, Tabriz, Khosroshahr, 1346 m, 37°58'28"N, 46°02'55"E, 21-30.August.2007, leg. Lotfalizadeh, Malaise trap (Fig. 1, ASh8).

Azarbaijan-e-Sharghi: 1♂, Sahand mountain, Kandovan, 2661 m, 37°45'47.7"N, 46°17'39.8"E, 1.September.2007, leg. Mozaffarian (Fig. 1, ASh10).

Dlabola (1981) reported this species from Zonuschay and Sufian (Fig. 1, ASh5, ASh 7).

###### Worldwide distribution.

East Palaearctic, Europe (Bulgaria, French mainland, Greek mainland, Italian mainland, Portuguese mainland, Sicily, Spanish mainland, Yugoslavia), Near East, North Africa (De Jong 2013).

##### Handianus bejbienkoi

Taxon classificationAnimaliaHemipteraCicadellidae

Dlabola, 1959

Handianus bejbienkoi : Emeljanov 1964: 523, fig. 188: 26–27.

###### Material examined.

Ardabil: 1♂, 10 km to Parsabad, 39°36'8.3"N, 47°48'45.5"E, 18.January.2007, leg. Mozaffarian (Fig. 1, A2).

Dlabola 1981 reported this species from Zonuschay and Maku (Fig. 1, ASh5, AG1).

###### Worldwide distribution.

East Palaearctic, Europe (Ukraine), Near East (De Jong 2013).

##### Hardya anatolica

Taxon classificationAnimaliaHemipteraCicadellidae

Zachvatkin, 1946*

###### Localities.

Marand (Dlabola 1981) (Fig. 1, ASh6).

###### Worldwide distribution.

Europe (Bulgaria, Greek mainland, Italian mainland, Romania, Yugoslavia), Near East (De Jong 2013).

##### Hardya iranicola

Taxon classificationAnimaliaHemipteraCicadellidae

Zachvatkin, 1946*

###### Localities.

Sufian (Dlabola 1981) (Fig. 1, ASh7).

###### Worldwide distribution.

Iran (Nast 1972).

##### Platymetopius chloroticus

Taxon classificationAnimaliaHemipteraCicadellidae

Puton, 1877*

###### Localities.

Sufian, Zonuschay (Dlabola 1981) (Fig. 1, ASh7, ASh5).

###### Worldwide distribution.

East Palaearctic, Europe (South Russia, Ukraine), Near East (De Jong 2013).

##### Platymetopius safavii

Taxon classificationAnimaliaHemipteraCicadellidae

Dlabola, 1971*

###### Localities.

Sufian (Dlabola 1981) (Fig. 1, ASh7).

###### Worldwide distribution.

Iran (Dlabola 1981).

##### Platymetopius shirazicus

Taxon classificationAnimaliaHemipteraCicadellidae

Dlabola, 1974*

###### Localities.

Marand (Dlabola 1981) (Fig. 1, ASh6).

###### Worldwide distribution.

Iran (Dlabola 1981).

#### Tribe: Chiasmini

##### Aconura jakowlefi

Taxon classificationAnimaliaHemipteraCicadellidae

Lethierry, 1876*

###### Localities.

Sufian, Zonuschay (Dlabola 1981) (Fig. 1, ASh7, ASh5).

###### Worldwide distribution.

East Palaearctic, Europe (South Russia), Near East (De Jong 2013).

##### Chiasmus conspurcatus

Taxon classificationAnimaliaHemipteraCicadellidae

(Perris, 1857)*

###### Localities.

Miyaneh-Siah chaman (Dlabola 1971) (Fig. 1, ASh15).

###### Worldwide distribution.

East Palaearctic, Europe (Albania, Austria (doubtful), Bulgaria, Canary Is., French mainland, Greek mainland, Italian mainland, Portuguese mainland, Romania, Sardinia, Sicily, Slovenia, Spanish mainland, Switzerland, Yugoslavia), Near East (De Jong 2013).

##### Doratura marandica

Taxon classificationAnimaliaHemipteraCicadellidae

Dlabola, 1981*

###### Localities.

Marand (Dlabola 1981) (Fig. 1, ASh6).

###### Worldwide distribution.

Iran (Dlabola 1981).

##### Doratura stylata

Taxon classificationAnimaliaHemipteraCicadellidae

(Boheman, 1847)

Doratura stylata : Le Quesne 1969: 67, figs 329–331; Biedermann and Niedringhaus 2009: 298.

###### Material examined.

Aradebil: 11♂♀, Sarein. Ardestan, 1700 m, 2.July.1997, leg. Barari & Mofidi (Fig. 1, A3).

Azarbaijan-e-Sharghi: 1♂, Bonab, 13.6 m, 37° 26'14.4"N, 045° 57'56.7"E, 27.August, 2007, leg. Mozaffarian & Nematian (Fig. 1, ASh9).

Azarbaijan-e-Sharghi: 9♂♀, Kandovan, 2645 m, 37° 45'45.8"N, 46° 17'40.5"E, 18.January.2008, leg. Mozaffarian. (Fig. 1, ASh10).

###### Worldwide distribution.

East Palaearctic, Europe (Albania, Austria, Belgium, Britain I., Bulgaria, Czech Republic, Danish mainland, Estonia, Finland, French mainland, Germany, Greek mainland, Hungary, Italian mainland, Latvia, Lithuania, Moldova, Norwegian mainland, Poland, Portuguese mainland, Romania, Russia Central, Russia North, South Russia, Slovakia, Slovenia, Spanish mainland, Sweden, Switzerland, The Netherlands, Ukraine, Yugoslavia), Near East, Nearctic region, North Africa (De Jong 2013).

This species is newly recorded from Iran.

##### Doraturopsis heros

Taxon classificationAnimaliaHemipteraCicadellidae

(Melichar, 1902)*

###### Localities.

Zonuschay, Marand (Dlabola 1981) (Fig. 1, ASh5, ASh6).

###### Worldwide distribution.

Europe (South Russia, Ukraine) (De Jong 2013).

#### Tribe: Cicadulini

##### Stenometopiellus iranicus

Taxon classificationAnimaliaHemipteraCicadellidae

Zachvatkin, 1946*

###### Localities.

Marand (Dlabola 1981) (Fig. 1, ASh6).

###### Worldwide distribution.

Iran (Dlabola 1981); Uzbekistan (Nast 1972).

#### Tribe: Goniagnathini

##### Goniagnathus brevis

Taxon classificationAnimaliaHemipteraCicadellidae

(Herrich-Schäffer, 1835)

Goniagnathus brevis : Emeljanov 1964: 501, fig. 180: 7, 8; Biedermann and Niedringhaus 2009: 283.

###### Material examined.

Azarbaijan-e-Sharghi: 1♂, Kaleibar, 1863 m, 38°52'13.5"N, 46°58'14.5"E, 2.September.2007, leg. Mozaffarian (Fig. 1, ASh3).

Abdollahi et al. (2013) reported this species from the above locality.

###### Worldwide distribution.

East Palaearctic, Europe (Albania, Austria, Belgium, Bulgaria, Cyprus, Czech Republic, European Turkey, French mainland, Germany, Greek mainland, Hungary, Italian mainland, Moldova, Poland, Portuguese mainland, Romania, South Russia, Sicily, Slovakia, Spanish mainland, Switzerland, The Netherlands, Ukraine, Yugoslavia), Near East, North Africa (De Jong 2013).

##### Goniagnathus guttulinervis

Taxon classificationAnimaliaHemipteraCicadellidae

(Kirschbaum, 1868)*

###### Localities.

Sufian (Dlabola 1981) (Fig. 1, ASh7).

###### Worldwide distribution.

Afro-tropical region, East Palaearctic, Europe (Balearic Is., Canary Is., French mainland, Greek mainland, Hungary, Italian mainland, Portuguese mainland, South Russia, Sardinia, Sicily, Spanish mainland, The Netherlands (doubtful), Ukraine), Near East, North Africa (De Jong 2013).

##### Goniagnathus minor

Taxon classificationAnimaliaHemipteraCicadellidae

Kusnezov, 1928*

###### Localities.

Miyaneh- Siah chaman (Dlabola 1971) (Fig. 1, ASh15).

###### Worldwide distribution.

Ukraine (Nast 1972).

#### Tribe: Hecalini

##### Hecalus glaucescens

Taxon classificationAnimaliaHemipteraCicadellidae

(Fieber, 1866)*

###### Localities.

Sufian (Dlabola 1981) (Fig. 1, ASh7).

###### Worldwide distribution.

East Palaearctic, Europe (Bulgaria, Cyprus, Greek mainland, Italian mainland, South Russia, Sicily, Slovakia, Spanish mainland, Ukraine, Yugoslavia), Near East, North Africa (De Jong 2013).

#### Tribe: Limotettigini

##### Limotettix striola

Taxon classificationAnimaliaHemipteraCicadellidae

(Fallén, 1806)

Limotettix striola : Emeljanov 1964: 529, fig. 190: 2–5; Biedermann and Niedringhaus 2009: 322.

###### Material examined.

Ardabil: 1♀, Moghan, Parsabad, 9.May.1969, leg. Abaii. (Fig. 1, A1)

Azarbaijan-e-Sharghi: 1♀, Tabriz, Gharachaman, 1600 m, 16.January.1976, leg. Boroumand & Pazouki (Fig. 1, ASh8).

Dlabola (1981) reported this species from Sufian (Fig. 1, ASh7).

###### Worldwide distribution.

East Palaearctic, Europe (Albania, Austria, Azores, Belgium, Britain I., Bulgaria, Cyprus, Czech Republic, Danish mainland, Estonia, Finland, French mainland, Germany, Greek mainland, Hungary, Ireland, Italian mainland, Latvia, Lithuania, Moldova, Norwegian mainland, Poland, Portuguese mainland, Romania, Russia Central, Russia North, South Russia, Slovakia, Slovenia, Spanish mainland, Sweden, Switzerland, The Netherlands, Ukraine, Yugoslavia), Near East, Nearctic region, North Africa (De Jong 2013).

#### Tribe: Macrostelini

##### Balclutha flavella

Taxon classificationAnimaliaHemipteraCicadellidae

Linnavuori, 1962*

###### Localities.

Zonuschay, Marand (Dlabola 1981) (Fig. 1, ASh5, ASh6).

###### Worldwide distribution.

Israel (Nast 1972).

##### Balclutha punctata

Taxon classificationAnimaliaHemipteraCicadellidae

(Fabricius, 1775)

Balclutha punctata : Emeljanov 1964: 507, fig. 182: 3; Biedermann and Niedringhaus 2009: 286.

###### Material examined.

Ardabil: 32♂♀, Heiran, 1527 m, 37°41'07.4"N, 48°23'57.4"E, 18.January.2007, Light trap, leg. Mozaffarian (Fig. 1, A4).

###### Worldwide distribution.

Australian region, East Palaearctic, Europe (Albania, Austria, Britain I., Bulgaria, Cyprus, Czech Republic, Danish mainland, Estonia, Finland, French mainland, Germany, Greek mainland, Hungary, Ireland, Italian mainland, Latvia, Lithuania, Moldova, Norwegian mainland, Poland, Russia Central, Russia North, South Russia, Sardinia, Sicily, Slovakia, Sweden, Switzerland, The Netherlands, Ukraine, Yugoslavia), Near East, Nearctic region, North Africa, Oriental region (De Jong 2013).

This species is newly recorded from northwestern Iran.

##### Balclutha rhenana

Taxon classificationAnimaliaHemipteraCicadellidae

Wagner, 1939*

###### Localities.

Marand (Dlabola 1981) (Fig. 1, ASh6).

###### Worldwide distribution.

East Palaearctic, Europe (Austria, Bulgaria, Czech Republic, Finland, Germany, Greek mainland (doubtful), Slovakia, Switzerland, The Netherlands, Yugoslavia) (De Jong 2013).

##### Macrosteles chobauti

Taxon classificationAnimaliaHemipteraCicadellidae

Ribaut, 1952

Macrosteles chobauti : Ribaut 1952: 48, figs 26–28.

###### Material examined.

Azarbaijan-e-Sharghi: 51♂♀, Kandovan, 2645 m, 37°45'45.8"N, 46°17'40.5"E, 18.January.2008, leg. Mozaffarian (Fig. 1, ASh10).

Abdollahi et al. (2014) also reported this species from the above locality.

###### Worldwide distribution.

Europe (Bulgaria, French mainland, Greek mainland) (De Jong 2013), France, Israel (Nast 1972).

##### Macrosteles fieberi

Taxon classificationAnimaliaHemipteraCicadellidae

(Edwards, 1889)*

###### Localities.

Sufian (Dlabola 1981) (Fig. 1, ASh7).

###### Worldwide distribution.

East Palaearctic, Europe (Austria, Britain I., Bulgaria, Czech Republic, Finland, French mainland, Germany, Greek mainland (doubtful), Ireland, Moldova, Norwegian mainland, Poland, Romania, South Russia, Slovakia, Sweden, Switzerland, The Netherlands, Ukraine, Yugoslavia), Near East, Nearctic region (De Jong 2013).

##### Macrosteles laevis

Taxon classificationAnimaliaHemipteraCicadellidae

(Ribaut, 1927)*

###### Localities.

Zonuschay, Maku (Dlabola 1981); Miynaeh-Zanjan, Miyaneh-Siah chaman, Tabriz-Shabestar (Dlabola 1971) (Fig. 1, ASh5, AG1, ASh16, ASh15, ASh7).

###### Worldwide distribution.

East Palaearctic, Europe (Albania, Austria, Belgium, Britain I., Bulgaria, Czech Republic, Danish mainland, Estonia, Finland, French mainland, Germany, Greek mainland, Hungary, Iceland, Italian mainland, Latvia, Lithuania, Moldova, Norwegian mainland, Poland, Romania, Russia Central, Russia North, South Russia, Slovakia, Sweden, Switzerland, The Netherlands, Ukraine, Yugoslavia), Near East, Nearctic region (De Jong 2013).

###### Comment.

Kheyri (1989) reported this species as a sugar beet pest in most sugar beet growing areas in Iran.

##### Macrosteles sexnotatus

Taxon classificationAnimaliaHemipteraCicadellidae

(Fallén, 1806)

Macrosteles sexnotatus : Emeljanov 1964: 507, fig. 182: 26, 27; Biedermann and Niedringhaus 2009: 288.

###### Material examined.

Azarbaijan-e-Sharghi: 30♂♀, Sahand mountain, Kandovan, 2661 m, 37°45'47.7"N, 46°17'39.8"E, 1.September.2007, leg. Mozaffarian (Fig. 1, ASh10).

Abdollahi et al. (2013) reported this species from the above locality.

###### Worldwide distribution.

East Palaearctic, Europe (Austria, Azores, Belgium, Britain I., Bulgaria, Canary Is., Cyprus, Czech Republic, Danish mainland, Estonia, Finland, French mainland, Germany, Greek mainland, Hungary, Iceland, Ireland, Italian mainland, Latvia, Lithuania, Madeira, Moldova, Norwegian mainland, Poland, Portuguese mainland, Romania, Russia Central, South Russia, Sardinia, Sicily, Slovakia, Spanish mainland, Sweden, Switzerland, The Netherlands, Ukraine, Yugoslavia), Near East, North Africa (De Jong 2013).

##### Macrosteles sordidipennis

Taxon classificationAnimaliaHemipteraCicadellidae

(Stål, 1858)

Macrosteles sordidipennis : Emeljanov 1964: 507, fig. 182: 36, 37; Biedermann and Niedringhaus 2009: 290.

###### Material examined.

Azarbaijan-e-Sharghi: 1♂, Sahand Mountain, Kandovan, 2661 m, 37°45'47.7"N, 46°17'39.8"E, 1.September.2007, leg. Mozaffarian (Fig. 1, ASh10).

###### Worldwide distribution.

East Palaearctic, Europe (Austria, Britain I., Czech Republic, Danish mainland, Finland, Germany, Hungary, Norwegian mainland, Poland, Russia North, Sweden, The Netherlands) (De Jong 2013).

This species is newly recorded from Iran.

#### Tribe: Opsiini

##### Concavifer marmoratus

Taxon classificationAnimaliaHemipteraCicadellidae

Dlabola, 1960*

###### Localities.

Zonuschay (Dlabola 1981) (Fig. 1, ASh5).

###### Worldwide distribution.

Iran, Israel, Kazakhstan, Tadzhikistan (Nast 1972).

##### Neoaliturus fenestratus

Taxon classificationAnimaliaHemipteraCicadellidae

(Herrich-Schäffer, 1834)*

###### Localities.

Tabriz (Dlabola 1981) (Fig. 1, ASh8).

###### Worldwide distribution.

East Palaearctic, Europe (Albania, Austria, Balearic Is., Belgium, Bulgaria, Canary Is., Cyprus, Czech Republic, Danish mainland, European Turkey, French mainland, Germany, Greek mainland, Hungary, Italian mainland, Latvia, Lithuania, Moldova, Poland (doubtful), Portuguese mainland, Romania, Russia Central, Russia North, South Russia, Sardinia, Sicily, Slovakia, Spanish mainland, Switzerland, The Netherlands, Ukraine, Yugoslavia), Near East, North Africa (De Jong 2013).

##### Neoaliturus haematoceps

Taxon classificationAnimaliaHemipteraCicadellidae

(Mulsant Rey, 1855)*

###### Localities.

Marand, Zonuschay, Sufian, Maku (Dlabola 1981) (Fig. 1, ASh6, ASh5, ASh7, AG1).

###### Worldwide distribution.

Afghanistan, Algeria, Austria, Canary Is., Cyprus, Czechoslovakia (Bohemia, Moravia, Slovakia), Egypt, France, German FR., Greece, Hungary, Iran, Italy (also Sardinia and Sicily), Jordan, Lebanon, Libya, Madeira Archipelago, Mongolia, Morocco, Poland, Romania, Spain, Syria, Tunisia, Turkey (Anatolia), Armenia, Azerbaijan, Georgia, Kazakhstan, Kirghizia, Moldavia, s.Russia, Turkmenia, Ukraine, Uzbekistan, Yugoslavia (Nast 1972).

###### Comment.

Kheyri (1989) reported this species as an economic pest on sugar beet from Isfahan, Kerman, Fars, Khorasan, Azarbaijan and Karaj.

##### Neoaliturus opacipennis

Taxon classificationAnimaliaHemipteraCicadellidae

(Lethierry, 1876)*

###### Localities.

Miyaneh-Zanjan, Bostanabad-Siah chaman, Siah chaman-Basmenj (Dlabola 1971) (Fig. 1, ASh16, ASh14, ASh13).

###### Worldwide distribution.

Europe (Cyprus, French mainland, Germany, Greek mainland, Italian mainland, South Russia, Sardinia, Sicily, Spanish mainland, Switzerland, Ukraine), Near East, North Africa (De Jong 2013).

###### Comment.

Kheyri and Alimoradi (1969) reported this species as a vector of curly top virus in Khorasan, Fars, Isfehan, Kerman, Ahvaz and Karaj.

##### Neoaliturus pulcher

Taxon classificationAnimaliaHemipteraCicadellidae

(Haupt, 1927)*

###### Localities.

Zonuschay (Dlabola 1981) (Fig. 1, ASh5).

###### Worldwide distribution.

Iran, Israel, Georgia, Kazakhstan, Tadzhikistan (Nast 1972).

##### Opsius cypriacus

Taxon classificationAnimaliaHemipteraCicadellidae

Lindberg, 1958*

###### Localities.

Zonuschay (Dlabola 1981) (Fig. 1, ASh5).

###### Worldwide distribution.

Europe (Cyprus, Greek mainland, Ukraine), Near East (De Jong 2013).

##### Opsius discessus

Taxon classificationAnimaliaHemipteraCicadellidae

(Horváth, 1911)*

###### Localities.

Zonuschay, Marand (Dlabola 1981) (Fig. 1, ASh5, ASh6).

###### Worldwide distribution.

East Palaearctic, Europe (South Russia), Near East (De Jong 2013).

##### Opsius pallasi

Taxon classificationAnimaliaHemipteraCicadellidae

(Lethierry, 1874)*

###### Localities.

Zonuschay, Marand (Dlabola 1981) (Fig. 1, ASh5, ASh6).

###### Worldwide distribution.

Europe (South Russia) (De Jong 2013).

##### Opsius scutellaris

Taxon classificationAnimaliaHemipteraCicadellidae

(Lethierry, 1874)*

###### Localities.

Zonuschay (Dlabola 1981) (Fig. 1, ASh5).

###### Worldwide distribution.

Afro-tropical region, East Palaearctic, Europe (Canary Is.), Near East, North Africa (De Jong 2013).

##### Pseudophlepsius binotatus

Taxon classificationAnimaliaHemipteraCicadellidae

(Signoret, 1880)*

###### Localities.

Zonuschay, Sufian (Dlabola 1981) (Fig. 1, ASh5, ASh7).

###### Worldwide distribution.

Europe (South Russia) (De Jong 2013).

#### Tribe: Paralimnini

##### Mogangella straminea

Taxon classificationAnimaliaHemipteraCicadellidae

Dlabola, 1957

Mogangella straminea : Emeljanov 1964: 541, fig. 194: 13, 14.

###### Material examined.

Azarbaijan-e-Sharghi: 1♂ 1♀, Marand, 12.July.2007, Light trap, leg. Lotfalizadeh (Fig. 1, ASh6).

###### Worldwide distribution.

East Palaearctic, Europe (Moldova, Ukraine), Near East (De Jong 2013).

This species is newly recorded from Iran.

##### Paramesus major

Taxon classificationAnimaliaHemipteraCicadellidae

Haupt, 1927*

###### Localities.

Sufian (Dlabola 1981) (Fig. 1, ASh7).

###### Worldwide distribution.

East Palaearctic, Europe (Austria, Bulgaria, Czech Republic, Germany (doubtful), Greek mainland, Hungary, Poland, South Russia, Yugoslavia), Near East (De Jong 2013).

##### Paramesus paludosus

Taxon classificationAnimaliaHemipteraCicadellidae

Ribaut, 1952*

###### Localities.

Sufian (Dlabola 1981) (Fig. 1, ASh7).

###### Worldwide distribution.

France, Italy, Kazakhstan, Moldavia, Ukraine (Nast 1972).

##### Psammotettix alienus

Taxon classificationAnimaliaHemipteraCicadellidae

(Dahlbom, 1850)

Psammotettix alienus : Ribaut 1952: 243, figs 579–580; Emeljanov 1964: 541, fig. 194: 8, 9; Biedermann and Niedringhaus 2009: 337.

###### Material examined.

Ardabil: 4♂♀, Moghan, 18.May.1978, leg. Abaii (Fig. 1, A1).

Azarbaijan-e-Sharghi: 3♂♀, Marand, 1610 m, 28.July.1976, leg. Broumand & Pazouki (Fig. 1, ASh6).

Azarbaijan-e-Sharghi: 1♀, Tabriz, Gharachaman, 1600 m, 16.July.1976, leg. Broumand & Pazouki (Fig. 1, ASh8).

Dlabola (1981) reported this species from Marand, Sufian and Zonuschay, Maku and in 1971 from Tabriz-Bostanabad, Miyaneh-Zanjan, Miyaneh-Siah chaman, Siah chaman-Basmenj (Fig. 1, ASh6, ASh7, ASh5, AG1, ASh12, ASh16, ASh15, ASh13).

###### Worldwide distribution.

East Palaearctic, Europe (Albania, Austria, Belgium, Bulgaria, Canary Is., Czech Republic, Danish mainland, Estonia, Finland, French mainland, Germany, Greek mainland, Hungary, Italian mainland, Latvia, Lithuania, Madeira, Moldova, Norwegian mainland, Poland, Portuguese mainland, Romania, Russia Central, Russia North, South Russia, Sicily, Slovakia, Slovenia, Spanish mainland, Sweden, Switzerland, Ukraine, Yugoslavia), Near East, Nearctic region, North Africa (De Jong 2013).

###### Comment.

Nematollahi and Khajehali (2000) reported this species as a vector for wheat dwarf virus on *Zea* (maize) in Isfahan.

##### Psammotettix pictipennis

Taxon classificationAnimaliaHemipteraCicadellidae

(Kirschbaum, 1868)*

###### Localities.

Miyaneh-Zanjan (Dlabola 1971); Marand, Sufian (Dlabola 1981) (Fig. 1, ASh16, ASh6, ASh7).

###### Worldwide distribution.

East Palaearctic, Europe (Austria, Bulgaria, Greek mainland, Hungary, Moldova, Romania, South Russia, Slovenia, Spanish mainland, Ukraine, Yugoslavia), Near East (De Jong 2013).

##### Psammotettix seriphidii

Taxon classificationAnimaliaHemipteraCicadellidae

Emeljanov, 1962

Psammotettix seriphidii : Emeljanov 1964: 539, fig. 193: 1, 2.

###### Material examined.

Ardabil: 87♂♀, 12 km to Khalkhal, 1998 m, 37°35'41.8"N, 48°37'54.3"E, 17.Janaury.2007, leg. Mozaffarian, light trap (Fig. 1, A5).

Ardabil: 1♂, 10 km to Parsabad, 80 m, 39°36'8.3"N, 47°48'45.5"E, 18.January.2007, leg. Mozaffarian (Fig. 1, A2).

Ardabil: 3♂♀, Moghan, 65 m, 39°37'30.7"N, 47°46'57.5"E, 19.January.2007, leg. Mozaffarian (Fig. 1, A1).

Azarbaijan-e-Sharghi: 6♂♀, AjabShir, Yaichi village, 1922 m, 37°35'27.2"N, 46°11'03.7"E, 15.January.2008, leg. Mozaffarian (Fig. 1, ASh11).

Azarbaijan-e-Sharghi: 2♂♀, Kaleibar, 1732 m, 38°54'25.2"N, 47°09'11.5"E, 3.September.2007, leg. Mozaffarian (Fig. 1, ASh2).

Azarbaijan-e-Sharghi: 1♂, Kandovan, 2645 m, 37°45'45.8"N, 46°17'40.5"E, 18.January.2008, leg. Mozaffarian (Fig. 1, ASh10).

Azarbaijan-e-Sharghi: 20♂♀, Kandovan, 1978 m, 37°44'15.8"N, 46°19'55.1"E, 18.January.2008, leg. Mozaffarian (Fig. 1, ASh10).

Azarbaijan-e-Sharghi: 4♂♀, Eskanlu, Aras river, 290 m, 39°12'13.4"N, 47°04'23.2"E, 3.September.2007, leg. Mozaffarian & Nematian (Fig. 1, ASh1).

Azarbaijan-e-Gharbi: 11♂♀, Uromieh, MirzaAbad, 1450 m, 21.July.1976, leg. Boroumand & Pazouki (Fig. 1, AG4).

###### Worldwide distribution.

Kazakhstan (Nast 1972).

This species is newly recorded from Iran.

##### Sorhoanus medius

Taxon classificationAnimaliaHemipteraCicadellidae

(Mulsant Rey, 1855)*

###### Localities.

Sufian (Dlabola 1981) (Fig. 1, ASh7)

###### Worldwide distribution.

Bulgaria, France, Italy, Switzerland, Altai Mts., Kazakhstan, Kirghizia, Russia, Siberia, Ukraine, Yugoslavia (Nast 1972).

#### Tribe: Phlepsiini

##### Phlepsius intricatus

Taxon classificationAnimaliaHemipteraCicadellidae

(Herrich-Schäffer, 1838)

Phlepsius intricatus : Emeljanov 1964: 516, fig. 185: 5, 6; Biedermann and Niedringhaus 2009: 305.

###### Material examined.

Azarbaijan-e-Sharghi: 2♂, Tabriz, Khosroshahr, 1346 m, 37°58'28"N, 46°02'55"E, 21-30.August.2007, leg. Lotfalizadeh, Malaise trap (Fig. 1, ASh8).

Dlabola (1981) reported this species from Zonuschay and in 1974 from Uromieh (Fig. 1, ASh5, AG3).

###### Worldwide distribution.

Europe (Albania, Austria, Balearic Is., Bulgaria, Czech Republic, European Turkey, French mainland, Germany, Greek mainland, Hungary, Italian mainland, Moldova, Portuguese mainland, Romania, South Russia, Sardinia, Sicily, Slovakia, Slovenia, Spanish mainland), Near East, North Africa (De Jong 2013).

#### Tribe: Scaphoideini

##### Anoplotettix magnificus

Taxon classificationAnimaliaHemipteraCicadellidae

Emeljanov, 1962*

###### Localities.

Sufian (Dlabola 1981) (Fig. 1, ASh7).

###### Worldwide distribution.

Azarbaijan, Georgia (Nast 1972).

### Subfamily: Dorycephalinae

#### Tribe: Eupelicini

##### Eupelix cuspidata

Taxon classificationAnimaliaHemipteraCicadellidae

(Fabricius, 1775)

Eupelix cuspidata : Ribaut 1952: 325, figs 868–871.

###### Material examined.

Azaibaijan-e-Sharghi: 4♂♀, Ajabshir, Yaichi village, 1922 m, 37°35'27.2"N, 46°11'03.7"E, 15.January.2008, leg. Mozaffarian (Fig. 1, ASh11).

Dlabola (1981) reported this species from Zonuschay, Marand and Sufian (Fig. 1, ASh5, ASh6, ASh7).

###### Worldwide distribution.

East Palaearctic, Europe (Albania, Austria, Balearic Is., Belgium, Britain I., Bulgaria, Canary Is., Corsica, Croatia, Cyprus, Czech Republic, Danish mainland, Estonia, Finland, French mainland, Germany, Greek mainland, Hungary, Ireland, Italian mainland, Latvia, Lithuania, Moldova, Norwegian mainland, Poland, Portuguese mainland, Romania, Russia Central, Russia North, South Russia, Sardinia, Sicily, Slovakia, Slovenia, Spanish mainland, Sweden, Switzerland, The Netherlands, Ukraine, Yugoslavia), Near East, North Africa (De Jong 2013).

##### Paradorydium aristidae

Taxon classificationAnimaliaHemipteraCicadellidae

(Zachvatkin, 1953)*

###### Localities.

Zonuschay, Maku (Dlabola 1981) (Fig. 1, ASh5, AG1).

###### Worldwide distribution.

East Palaearctic, Europe (South Russia, Ukraine), Near East (De Jong 2013).

### Subfamily: Iassinae

#### Tribe: Iassini

##### Batracomorphus irroratus

Taxon classificationAnimaliaHemipteraCicadellidae

Lewis, 1834*

###### Localities.

Marand (Dlabola 1981) (Fig. 1, ASh6).

###### Worldwide distribution.

East Palaearctic, Europe (Albania, Austria, Belgium, Britain I., Bulgaria, Czech Republic, Danish mainland, French mainland, Germany, Greek mainland, Hungary, Italian mainland, Lithuania, Moldova, Poland, South Russia, Slovakia, Switzerland, Ukraine, Yugoslavia), Near East (De Jong 2013).

### Subfamily: Idiocerinae

#### Tribe: Idiocerini

##### Rhytidodus resaicus

Taxon classificationAnimaliaHemipteraCicadellidae

Dlabola, 1974*

###### Localities.

Uromieh (Dlabola 1974) (Fig. 1, AG3)

### Subfamily: Macropsinae

#### Tribe: Macropsini

##### Hephathus unicolor

Taxon classificationAnimaliaHemipteraCicadellidae

(Lindberg, 1926)*

###### Localities.

Zonuschay (Dlabola 1981) (Fig. 1, ASh5).

###### Worldwide distribution.

East Palaearctic, Europe (Romania (doubtful), South Russia (doubtful), Ukraine (doubtful), Yugoslavia (doubtful)), Near East (De Jong 2013).

##### Hephathus freyi

Taxon classificationAnimaliaHemipteraCicadellidae

(Fieber, 1868)*

###### Localities.

Siah chaman-Miyaneh (Dlabola 1971) (Fig. 1, ASh15).

###### Worldwide distribution.

Europe (Balearic Is., Bulgaria, French mainland, Greek mainland (doubtful), Italian mainland, Portuguese mainland, Sicily, Slovakia, Spanish mainland, Yugoslavia), Near East, North Africa (De Jong 2013).

### Subfamily: Typhlocybinae

#### Tribe: Empoascini

##### Empoasca punjabensis

Taxon classificationAnimaliaHemipteraCicadellidae

Singh-Pruthi, 1940*

###### Localities.

Zonuschay, Maku (Dlabola 1981); Siah chaman-Miyaneh, Miyaneh-Zanjan, Tabriz-Shabestar, Uromieh-Sarv (Dlabola 1971) (Fig. 1, ASh5, AG1, ASh15, ASh16, ASh7, AG2).

###### Worldwide distribution.

Europe (Bulgaria, French mainland, Greek mainland, South Russia, Ukraine, Yugoslavia), Near East, Oriental region (De Jong 2013).

###### Comment.

Kheyri (1989) reported this species as an economic pest on sugar beet from Isfahan, Kerman, Fars and Karaj.

##### Kyboasca bipunctata

Taxon classificationAnimaliaHemipteraCicadellidae

(Oshanim, 1871)*

###### Localities.

Miyaneh- Siah chaman, Tabriz-Shabestar, Miyaneh-Zanjan (Dlabola 1971); Sufian (Dlabola 1981) (Fig. 1, ASh15, ASh7, ASh16, ASh7).

###### Worldwide distribution.

East Palaearctic, Europe (Austria, Bulgaria, Czech Republic, Danish mainland, Finland, Germany, Hungary, Italian mainland, Moldova, Poland, Romania, South Russia, The Netherlands, Ukraine, Yugoslavia), Near East, Nearctic region (De Jong 2013).

#### Tribe: Erythroneurini

##### Tamaricella ribauti

Taxon classificationAnimaliaHemipteraCicadellidae

(Zachvatkin, 1947)*

###### Localities.

Zonuschay (Dlabola 1981) (Fig. 1, ASh5).

###### Worldwide distribution.

Europe (Crete, South Russia, Ukraine) (De Jong 2013).

##### Tamaricella tamaricis

Taxon classificationAnimaliaHemipteraCicadellidae

(Puton, 1872)*

###### Localities.

Miyaneh-Zanjan, Miyaneh-Siah chaman (Dlabola 1971) (Fig. 1, ASh16, ASh15).

###### Worldwide distribution.

Europe (Bulgaria, Crete, Cyclades Is., Cyprus, French mainland, Greek mainland, Italian mainland, Romania, South Russia, Sardinia, Sicily, Spanish mainland, Ukraine) (De Jong 2013).

##### Zyginidia pullula

Taxon classificationAnimaliaHemipteraCicadellidae

(Boheman, 1845)*

###### Localities.

Marand (Dlabola 1981) (Fig. 1, ASh6).

###### Worldwide distribution.

Europe (Albania, Austria, Bulgaria, Corsica (doubtful), Czech Republic, Danish mainland, Finland, French mainland, Germany, Greek mainland, Hungary, Italian mainland, Romania, Slovakia, Spanish mainland, Sweden, Switzerland, Ukraine, Yugoslavia), Near East (De Jong 2013).

##### Zyginidia sohrab

Taxon classificationAnimaliaHemipteraCicadellidae

Zachvatkin, 1947*

###### Localities.

Miyaneh-Zanjan, Siah chaman-Miyaneh, Uromieh-Sarv (Dlabola 1971) (Fig. 1, ASh16, ASh15, AG2).

###### Worldwide distribution.

Europe (Cyprus, Greek mainland, South Russia, Ukraine), Near East (De Jong 2013).

#### Tribe: Typhlocybini

##### Edwardsiana rosae

Taxon classificationAnimaliaHemipteraCicadellidae

(Linné, 1758)*

###### Localities.

Siah chaman-Miyaneh (Dlabola 1971) (Fig. 1, ASh15).

###### Worldwide distribution.

East Palaearctic, Europe (Austria, Belgium, Britain I., Bulgaria, Cyprus, Czech Republic, Danish mainland, Estonia, Finland, French mainland, Germany, Greek mainland, Hungary, Ireland, Italian mainland, Latvia, Moldova, Norwegian mainland, Poland, Romania, Russia Central, Russia North, South Russia, Sicily, Slovakia, Spanish mainland, Sweden, Switzerland, The Netherlands, Ukraine, Yugoslavia), Near East, Nearctic region, Oriental region (De Jong 2013).

#### Subfamily: Ulopinae

##### Tribe: Ulopini

###### Ulopa trivia

Taxon classificationAnimaliaHemipteraCicadellidae

Germar, 1821*

####### Localities.

Marand (Dlabola 1981) (Fig. 1, ASh6).

####### Worldwide distribution.

Albania, Austria, Belgium, Bulgaria, Cyprus, Czechoslovakia (Bohemia, Moravia, Slovakia), Denmark, France, German DR, German FR, Great Britain (England), Greece, Hungary, Italy, Palestine, Poland, Portugal, Romania, Spain, Turkey (Anatolia), Azerbaijan, Armenia, Georgia, Moldavia, Russia, Ukraine, Yugoslavia (Nast 1972).

## Supplementary Material

XML Treatment for Agallia firdausica

XML Treatment for Anaceratagallia laevis

XML Treatment for Austroagallia sinuata

XML Treatment for Aphrodes bicinctus

XML Treatment for Cicadella viridis

XML Treatment for Conosanus obsoletus

XML Treatment for Eohardya miyaneha

XML Treatment for Euscelis alsius

XML Treatment for Handianus bejbienkoi

XML Treatment for Hardya anatolica

XML Treatment for Hardya iranicola

XML Treatment for Platymetopius chloroticus

XML Treatment for Platymetopius safavii

XML Treatment for Platymetopius shirazicus

XML Treatment for Aconura jakowlefi

XML Treatment for Chiasmus conspurcatus

XML Treatment for Doratura marandica

XML Treatment for Doratura stylata

XML Treatment for Doraturopsis heros

XML Treatment for Stenometopiellus iranicus

XML Treatment for Goniagnathus brevis

XML Treatment for Goniagnathus guttulinervis

XML Treatment for Goniagnathus minor

XML Treatment for Hecalus glaucescens

XML Treatment for Limotettix striola

XML Treatment for Balclutha flavella

XML Treatment for Balclutha punctata

XML Treatment for Balclutha rhenana

XML Treatment for Macrosteles chobauti

XML Treatment for Macrosteles fieberi

XML Treatment for Macrosteles laevis

XML Treatment for Macrosteles sexnotatus

XML Treatment for Macrosteles sordidipennis

XML Treatment for Concavifer marmoratus

XML Treatment for Neoaliturus fenestratus

XML Treatment for Neoaliturus haematoceps

XML Treatment for Neoaliturus opacipennis

XML Treatment for Neoaliturus pulcher

XML Treatment for Opsius cypriacus

XML Treatment for Opsius discessus

XML Treatment for Opsius pallasi

XML Treatment for Opsius scutellaris

XML Treatment for Pseudophlepsius binotatus

XML Treatment for Mogangella straminea

XML Treatment for Paramesus major

XML Treatment for Paramesus paludosus

XML Treatment for Psammotettix alienus

XML Treatment for Psammotettix pictipennis

XML Treatment for Psammotettix seriphidii

XML Treatment for Sorhoanus medius

XML Treatment for Phlepsius intricatus

XML Treatment for Anoplotettix magnificus

XML Treatment for Eupelix cuspidata

XML Treatment for Paradorydium aristidae

XML Treatment for Batracomorphus irroratus

XML Treatment for Rhytidodus resaicus

XML Treatment for Hephathus unicolor

XML Treatment for Hephathus freyi

XML Treatment for Empoasca punjabensis

XML Treatment for Kyboasca bipunctata

XML Treatment for Tamaricella ribauti

XML Treatment for Tamaricella tamaricis

XML Treatment for Zyginidia pullula

XML Treatment for Zyginidia sohrab

XML Treatment for Edwardsiana rosae

XML Treatment for Ulopa trivia
